# Interactively illustrating polymerization using three-level model fusion

**DOI:** 10.1186/1471-2105-15-345

**Published:** 2014-10-14

**Authors:** Ivan Kolesar, Julius Parulek, Ivan Viola, Stefan Bruckner, Anne-Kristin Stavrum, Helwig Hauser

**Affiliations:** Department of Informatics, University of Bergen, N-5020 Bergen, Norway; Vienna University of Technology, Vienna, Austria

**Keywords:** Biochemical visualization, L-system modeling, Multi-agent modeling, Visualization of physiology, Polymerization

## Abstract

**Background:**

Research in cell biology is steadily contributing new knowledge about many aspects of physiological processes, both with respect to the involved molecular structures as well as their related function. Illustrations of the spatio-temporal development of such processes are not only used in biomedical education, but also can serve scientists as an additional platform for in-silico experiments.

**Results:**

In this paper, we contribute a new, three-level modeling approach to illustrate physiological processes from the class of polymerization at different time scales. We integrate physical and empirical modeling, according to which approach best suits the different involved levels of detail, and we additionally enable a form of interactive steering, while the process is illustrated. We demonstrate the suitability of our approach in the context of several polymerization processes and report from a first evaluation with domain experts.

**Conclusion:**

We conclude that our approach provides a new, hybrid modeling approach for illustrating the process of emergence in physiology, embedded in a densely filled environment. Our approach of a complementary fusion of three systems combines the strong points from the different modeling approaches and is capable to bridge different spatial and temporal scales.

**Electronic supplementary material:**

The online version of this article (doi:10.1186/1471-2105-15-345) contains supplementary material, which is available to authorized users.

## Background

Polymers are macromolecules that are composed of many smaller molecules, known as monomers. Polymers with different structure and monomer composition have a broad range of different physical properties, like solution viscosity, melt viscosity, solubility, stiffness, and more. Well-known examples of polymers are proteins and the DNA, which play important roles in everyday life. Polymerization is the biochemical process of polymer formation. During polymerization, monomers react with each other to form a macromolecular structure. As polymers are essential components of biological processes, polymerization occurs constantly within the cells of every living organism.

Even though major advances in recent biological and biochemical research greatly extend our knowledge about polymerization, still much remains unknown. With respect to the involved molecular structures, for example, not all of them have been crystallized to derive a better understanding of their spatial structure. Also much remains unknown regarding their physiological function. This naturally inherent uncertainty is one important reason for why it is challenging, for students as well as for professionals from different fields, to form an appropriate mental model of physiological processes.

In order to effectively communicate such processes, it is essential to consider both their spatial and temporal characteristics as well as their multi-scale nature. Polymerization, for example, ranges spatially from molecules to macromolecules and temporally from nanoseconds (monomer movement) to seconds (overall process of polymerization). It is also not feasible to model the entire physiological processes by just considering the principal laws of physics on the atomic level – we need different models at different levels of details. Moreover, the process of polymerization strongly depends on the properties of the environment such as the concentration of the reacting substances.

In recent years, we have seen a growing number of artistic illustrations of various aspects of cell biology [1, 2] and we have also observed some selected efforts to, at least partially, support the usually cumbersome, manual illustration process with computational tools. However, for a better understanding and for a more effective communication of physiological processes, visualization in the form of static images or animations is often not enough. One should, for example, see the dependence of such a process on its environment and experiment with the interactions between the process and its environment. How will the structure emerge if there are not enough building substances? How do spatial constrains influence branching patterns? An interactive system capable of answering such questions can greatly help to comprehend the process of polymerization and even be an environment for generating or even testing new hypotheses.

For answering the above mentioned questions, a suitable modeling and visualization approach for the interactive illustration of polymerization should satisfy the following requirements:

- It needs to **capture emergence**, i.e., it should be capable of representing the overall process of emergence and its sub-processes, for example, the binding of monomers and branching.
- It needs to represent the **temporal development**, i.e., it has to communicate the time-dependent and dynamic nature of the process.
- The **multi-scale** nature of the process needs to be captured in both space and time.
- **Interactivity** is essential and the user should be able to modify the environment and immediately see the results.
- Even if based on empirical modeling approaches, the illustration must be **sufficiently biochemically correct**.

In this paper, we present a new, three-level modeling and visualization approach, which fulfills the above described requirements. A starting point for our research was the observation that polymerization is physiologically characterized by biochemical processes at different time scales (from nanoseconds to seconds) and that we were aiming at an approach which should be truthful to these different time scales.

The smallest time scales, which we intended to capture with our approach, are those that correspond to the diffusion-based movement of monomers nearby the active end of a polymer and the growing of the polymer due to individual monomers that bind to the polymer. Considering the advantages and disadvantages of different modeling approaches (as discussed in the Related work section), we concluded that an agent-based system would be best suited to capture the stochastic characteristic of the movement of the monomers. Also setting the different parameters for the agent based system allows us to set different behavior based at which time scale is currently visualized.

On the other end of the temporal scale space, we intended to capture the entire growth process of a polymer – a process which is many orders of magnitudes slower than the diffusion-based movement of the monomers. We understand that these polymerization processes (at a larger time scale) are much more deterministic in terms of their development. Therefore, it is appropriate to model the process at this level by means of an L-system (this is also in line with many other cases of biological growth, like plant growth [3]- [5], which regularly are modeled the same way).

To realize a solution which is capable of representing both of these aspects, we devised an approach which integrates both modeling concepts. We find it reasonably straight-forward to formulate rules for an L-system so that it models the overall growth of a polymer. We link – via a communication system (see the Communication & process specification section for more details) – the agent-based system to the L-system so that certain rewriting rules of the L-system – in particular those, which correspond to the binding of a monomer to the polymer – only complete, if they are supported by the linked agent-based system.

Furthermore, we intended to also enable a minimum amount of interactive steering – at least to the degree that the user can influence the environmental conditions of the polymerization process to a certain degree. To achieve this, we couple the agent-based system with another modeling layer, i.e., a density-based modeling layer (here called “system of densities”, SOD). On this layer, we only consider the overall densities of all involved building blocks (mostly the monomers). At any time, parallel to the overall modeling process, it influences the agent-based system so that the number of agents in the multi-agent system corresponds, as good as possible, with the corresponding densities in the SOD. By interactively modifying selected densities in the SOD, the user can thereby, to a certain degree, steer the polymerization process.

After we first discuss related work in the following, we then go into more technical details with respect to our solution. We also report from an evaluation which we conducted together with several domain experts.

## Related work

As mentioned above, our work is based on a fusion of three different modeling techniques, i.e., an L-system, an agent-based system, and a system of densities. In the following, we comment on the state of the art with respect to all of these individual approaches, as well as on previous attempts to extend them.

### L-systems

Lindenmayer systems [6] are a broadly used modeling approach for the development of linear and branching structures, built from discrete modules. An L-system can be seen as a formal, parallel rewriting grammar. It consists of an alphabet of symbols, a collection of rules that expand symbols into new symbols, or strings of symbols, an initial string, called axiom, and a mechanism for translating the generated string into an according geometric structure. Since the introduction of L-systems in the late 1960s, many extensions to the original approach were proposed, such as *stochastic*, *context-sensitive* and *parametric* L-systems, many of which are well described in a book by Prusinkiewicz and Lindenmayer [3].

Originally, L-systems lacked one important aspect of structural modeling, which is the interaction between the structure and its environment. The first extension that related L-systems to an environment as an affecting factor, were *parametric* L-systems [3, 7]. Here, every symbol is extended by its own parameter space, that is applied and changed by the production rules.

An *environmentally-sensitive* L-system [8] contains local, rather than global, properties of the environment that affect the model. This concept is based on query symbols, which return the position and orientation of the current, graphically interpreted symbol, in the given coordinate system. These parameters are passed as arguments to user-defined functions which then return local properties of the environment for the inquired location.

A more general approach for the communication between the model and the environment was introduced in *open* L-systems [4]. This technique extends environmentally-sensitive L-systems by using a special symbol for bidirectional communication with the environment. The environment is no longer represented as a simple function, but becomes an active process that may react to the information from the model. Open L-systems were used for modeling the development of different structures such as ecosystems [4, 9], cities [10], proteins folding [11], plants, trees and roots [5, 12], or even fire [13].

In our case, we find L-systems only partially suitable. While we, on the one hand, find them useful to represent the large-scale aspects of polymerization, their utility is, on the other hand, also limited, since they cannot intrinsically capture crucial small-scale characteristics of polymerization – in particular, the interaction of many individual actors (most importantly, the monomers and their behavior). Strengths and weaknesses of L-systems, with respect to modeling an illustration of polymerization, are shown in Table 1.

**Table 1:** **Selected strengths and weaknesses of L-systems vs. agent-based systems**

Modeling approach	Strengths	Weaknesses
**L-systems**	Suitable for modeling structures from empirical knowledge.	Limitations writing the creation of structure from stochastically behaved individual entities.
**Agent-based systems**	Ability to simulate a stochastic environment.	The global effect, resulting from the interaction of the individuals, is quite unpredictable.

### Agent-based systems

In contrast to L-systems, agent-based modeling [14] is centered around multiple autonomous entities called agents. Agents are computing elements with two important capabilities [15]. Firstly, they are capable of autonomous action, i.e., they can act independently in order to satisfy their designed objectives. Secondly, they are capable of interacting with other agents. An agent’s behavior is defined to achieve an individual or collective objective.

This modeling approach provides a natural metaphor for understanding and building a wide range of systems, such as social systems, biological systems, economics, traffic or transportation systems that feature many independent actors which drive the system’s global behavior.

In the context of emergent phenomena, agent-based systems have been employed in modeling molecular self-assembly [16, 17] and intracellular interactions [18, 19].

As agent-based systems model a global behavior through the interaction of individual entities, they are well suited for the purpose of modeling the crowded environment of the cell. However, a major drawback is that the global effect resulting from the interaction of the individual agents is very difficult to control and steer. In our case, we find agent-based modeling suitable for the small scale of polymerization, i.e., the movement of the monomers, etc., while we require more control over the modeling when considering the process at a larger scale.

### Integrated approaches

As shown in Table 1 both L-systems and agent-based modeling have strengths and weaknesses. Naturally, one thinks about the combination of both concepts to get the advantages of both approaches while mitigating their disadvantages. One way of integrating both approaches, researched by von Mammen, is *swarm grammars* [20, 21]. Swarm grammars were developed as an integrated representation of artificial crowds and a developmental model. In this approach, the L-system doesn’t hold the information about a structure, but about the agents’ states in the environment and is the deterministic tool for the evolution of the agents over time. The usefulness of such an approach was exemplified in the generation of the 3D geometry from the agents’ states [22] and the application of this method to architectural design [23]. However, with this modeling approach the graphical representation describes the development of the crowd, not the development of the structure. Moreover this approach doesn’t provide a modeling solution for bidirectional communication between the structure and the agents and is therefore not suitable for the interactive illustration of polymerization.

Other modeling approaches are based on the combination of rule-based and particle-based reaction and diffusion modeling [24, 25]. In these approaches the resulting molecular structures are represented as a graph, where each node is an elementary unit, for example, a simple molecule or a monomer. The molecules are defined as spatial particles and their behavior in the environment is described by molecular dynamics and reaction rules. The result of the combinations of the allowed interactions and the geometric requirements is a stochastically built molecule. These modeling approaches are using different visualization software (SRSim [24], ZygCell3D [26]), which provides direct visualization of the modeled polymerization.

In our modeling approach, we are introducing the probabilistic variability, i.e., the resulting molecular structure is not predetermined. With the L-system, our approach is capable of representing not only information about the current structure, but also information about processes that are currently associated with it. Furthermore, we know that the time scales between the overall process of creation of the structure (seconds) and the movement of a single independent molecule in the environment (nanoseconds) are largely different. We address these time scale differences by the possibility to interactively change the current time scale and the ability to switch between them. This helps to comprehend the creation of the structure and the relation between different time scales of the process. Also, for experiments, our solution provides steering of the simulation by changing the density (concentration) of the molecules in the environment. On top of that our solution provides a tool for changing the rules that define processes (reactions) during the simulation. Our approach provides a direct 3D visualization of the processes, but we can easily encode additional information in the visualized structure, for example the uncertainty of the creation of branches.

## Methods

**Figure 1:**
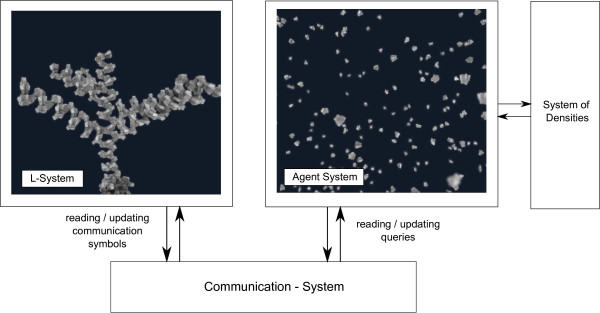
**The overview of our system.** An overall process is controlled by the L-System. Communication between L-System and Agent System is performed through the communication symbol that, using the Communication system, is transferred to the Agent System in the form of queries. Results from the queries are written back to the communication symbols and processed by L-System’s production rules. The system of densities provides means to change the amount of agents in the environment.



Our solution is composed of several different sub-systems (see Figure 1), which are mutually synchronized with each other. The simulation runs in a cuboid domain of changeable dimensions with a time step of length *Δt*.

**Figure 2:**
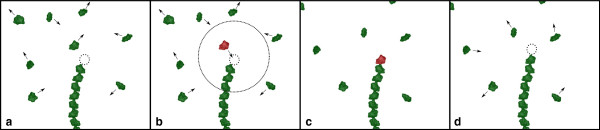
**Illustration of several simulation steps.** After the initial configuration **(a)**, a communication symbol was generated, which attracts nearby agents **(b)**. When the agent arrives at the binding site, it is attached to the structure and the communication symbol is terminated **(c)**. Afterwards a new communication symbol is created by production rules and is again attracting nearby agents **(d)**.

As depicted in Algorithm 1, the simulation starts with setting the simulation time *t*, the current delta time of the simulation *Δt* and the initialization of the simulation systems: the L-system (LS), the communication system (CS), the agent-based system (AS) and the system of densities (SOD). The basic cycle, shown also in Figure 2, is composed of the following steps:

- The L-system is evaluated, which involves processing of the communication with the monomers and growing of the polymer if a new monomer binds to the growing end. (Line 8)
- The L-system structure is visualized. (Line 9)
- The SOD verifies the current densities and communicates the required changes to the agent-based system. (Line 10)
- The communication system firstly evaluates on which time scale the simulation is currently running. This is done by the evaluation of the function *P*(*Δt*), which is described in more detail in the Communication & process specification subsection. If *P*(*Δt*)<*timeScaleTresh*, i.e., the time delta is relevant for monomer motion, the communication system transfers communication parameters from the L-system to the agent-based system and vice versa. In the case that *P*(*Δt*)>*timeScaleTresh*, the growth is computed from the probability function *P*(*Δt*). (Line 11)
- If *P*(*Δt*)<*timeScaleTresh*, meaning the simulation is in the monomer motion time scale, the agent-based system is evaluated and visualized. (Lines 12, 13, 14)

In the following subsections we provide a more detailed description of the mentioned components.

### L-System

The L-system consists of an ordered triplet *L*=〈*A*,*ω*,*P*〉, where *A* denotes an alphabet, *ω* is a non-empty word called axiom and *P* is a finite set of production rules. The axiom  defines the initial development of a polymer of size *n* in the simulation.

The symbols of the alphabet *A* are divided into four semantic categories: *Binding*, *Structure*, *End*, and *Communication* symbols. A *Structure* symbol represents a monomer and holds information about the monomer type and its geometry. A *Binding* symbol represents the binding relation between two monomers and holds information whether the binding point is a start of the new branch. The end of a branch is encoded by the *End* symbol. These symbols describe the structural aspects of a polymer in the L-system.

Processes are represented by *Communication* symbols. A communication symbol has the role of a bidirectional bridge between the L-system and the agent-based system through the communication system. It is defined by *C*(*O*,*Type*,*t*,*r*), where *O* identifies the process, e.g., growing or branching, *Type* is the identification of the agent type the process is connected to, for example, *t* is the process lifespan and *r* encodes the result of the process. For example, the communication symbol *C*(*binding*,*glucose*,5.0,*r*) queries information about the process binding the glucose molecule and expects the result in parameter *r*. Communication symbols have a global parameter *t*_*max*_ defining the maximum allowed time that the process can take. If the process is about to take longer, it is terminated.

A production rule from *P* has the following format [4]:


where *id* is the rule identifier (label), *predecessor* is a symbol that will be replaced by the *successor* symbol, but only if *condition* is evaluated as *true*. The *probability* part represents a chance value that this production rule will happen at all.

The L-system has two important phases: derivation and interpretation. The derivation step is the rewriting process: . In each step, the production rules *P* replace all predecessor symbols *ω*_*i*_ by successor symbols, generating a new string *ω*_*i*+1_.

**Figure 3:**
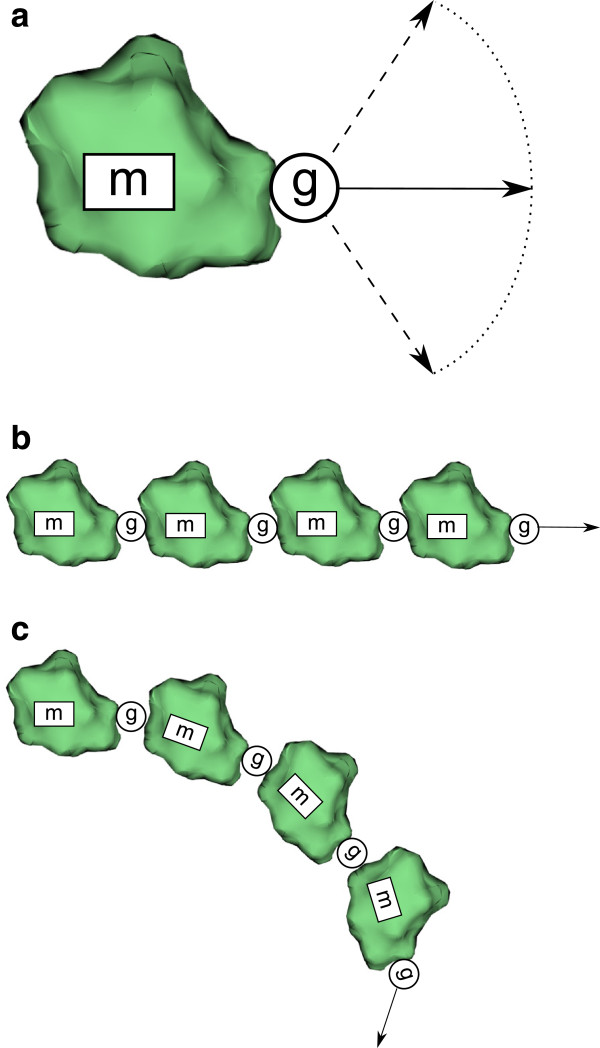
**Illustration of building the polymer from symbols of the current L-system state.** **(a)** The backbone of the interpretation process are structural (symbol m represents monomer) and binding symbols (symbol g represents binding between two monomers in predefined direction). The overall look of the final polymer is dependent on the visualization of structural symbols and their placement based on the property of binding symbol. If the binding symbol is defining linear conformation between the two monomers, linear structure will assemble **(b)**. The conformation with different orientation can create helices of different radii and helicities **(c)**.

The derivation step is followed by an interpretation step that transforms a string of symbols into a 3D geometrical representation. During the interpretation step, the string is read from left to right by an interpreter. The interpreter stores its spatial position *I*_*pos*_ (vector) and orientation *I*_*ori*_ (quaternion). These variables are initialized at the beginning of the interpretation step by the position and orientation of the polymer starting point. When the interpreter reads a structure symbol, then it places the geometry specified by it into the scene according to the current *I*_*pos*_ and *I*_*ori*_. When the interpreter reads a binding symbol, it updates its position and orientation as follows:


where *Bin*_*pos*_ and *Bin*_*ori*_ are the binding position (vector) and orientation (quaternion) from the binding symbol. Using this transformation the system can create the geometric representation of the whole polymer (Figure 3). Also, during this interpretation step the position and orientation parameters of the communication symbols are updated with the *I*_*pos*_ and *I*_*ori*_ of the current state.

Essentially, the evaluation of the L-system depicts the development of the polymer growth. First, the *r* parameters of the communication symbols are filled with values, retrieved from the communication system. Next, the derivation and interpretation phases are applied.

For example, let us define an L-system with the axiom *C*(*grow*,*molecule*,0,∅) and the following production rules:


The *t*_*max*_ parameter is an empirically chosen time limitation of the *grow* process. In the beginning of the L-system evaluation the *t* and *r* parameters of the C symbol are retrieved from the communication system. Afterwards, in the derivation phase, the production rules are applied.

Only the rules with the same predecessor and correct predecessor parameters are applied. For example, in a case when *t*=0.05 and *r*=∅, during the derivation step no production rules can be applied since both conditions *r*≠∅ and *t*>*t*_*max*_ of the rules *p*_1_ and *p*_2_ are not met. In this case, the L-system’s string is left unchanged.

When the agent system, through the communication system, returns values *t*=0.05 and *r*=*molecule*, the derivation step applies rule *p*_1_ and produces the new string *ω*=*mC*(*grow*,*molecule*,0,∅) with a new symbol *m*, and the communication symbol is replaced by *C*(*grow*,*molecule*,0,∅). This means that the growing process has finished and a new process of growing is created at the end of the structure.

If the process takes too long for values *t*=5.05 and *r*=∅, rule *p*_2_ is applied, rewriting the communication symbol to the end symbol; i.e., the growing process of the current branch is terminated.

### Communication & process specification

The information exchange between the L-system and the agent-based system is realized through the communication system. The behavior of this system depends on the current time scale of the simulation.

If the simulation is running in the time scale of monomer motion, the communication system retrieves the processes parameters from the L-system and transports them in a form of queries to the agent-based system. After the simulation step of the agent-based system, the communication system retrieves the results of the agent-based system queries and feeds them to the communication symbol of the L-system.

The query is represented as a *Q*(*pos, ori, type, time, result*). The position, orientation and type parameters are retrieved from the L-system interpreter; and copied into *pos*, *ori* and *type*. The agent-based system updates the parameters *time* and *result*. The *result* is an agent type and the system fills this value if and only if an agent of the specified type reaches the position *pos* with the orientation *ori*.

**Figure 4:**
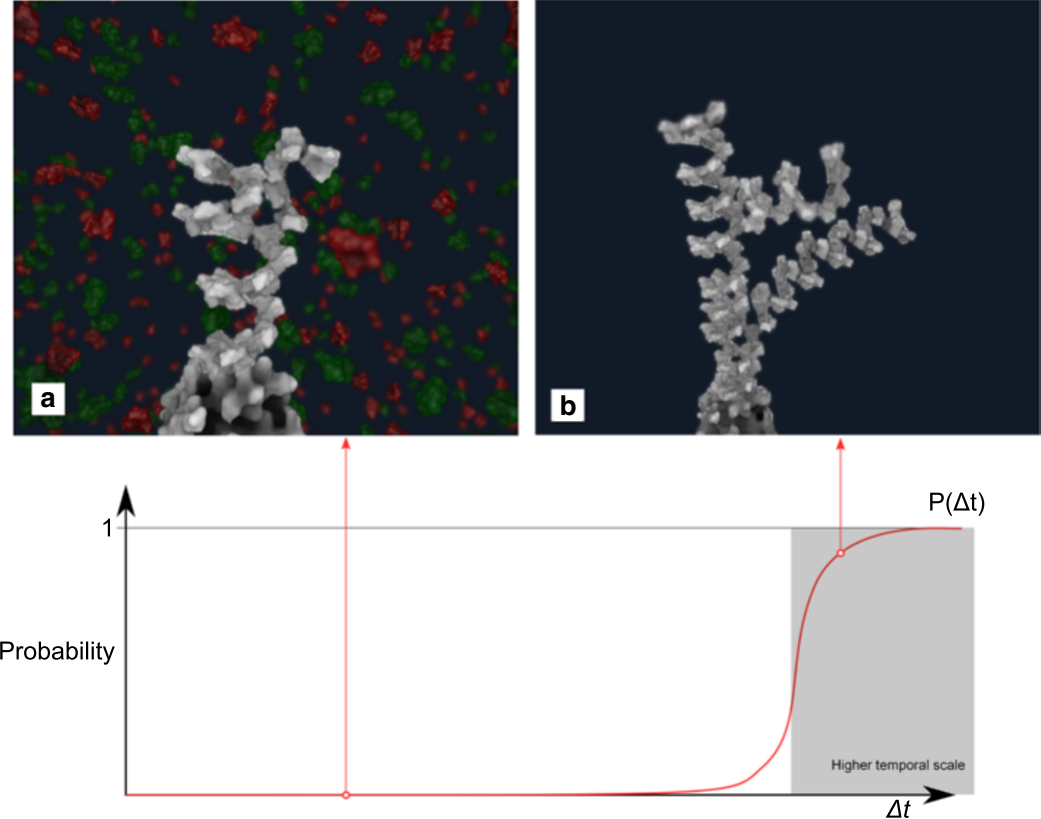
**An example of the probability function** ***P*** **(** ***Δ*** ***t*** **).** When *P*(*Δ* *t*)=0 the simulation runs in the lower time scale **(a)**, while for higher values it runs in the time scale of the entire process **(b)**.

On the other hand, if the simulation runs on the time scale of the whole process, the agent-based system does not participate in the communication. Instead, the communication system applies the function *P*(*Δt*), computing a probability of the temporal event for the *result* of query *Q*. The function *P*(*Δt*) is a probabilistic description of the process with respect to *Δt*. An example of this function is shown in Figure 4. The function *P* returns 0 if the *Δt* is lower then the threshold for time scale switching, and a value from 0 to 1 for a larger value of *Δt*. The assignment of the agent-based system and *P*(*Δt*) to the *result* parameter is described by the following equation:

where the function *P*(*Δt*) is the aforementioned probability function. The first term *P*(*Δt*)*d*_*type*_(*t*)*a*_*type*_ denotes the return value if the simulation happens at a larger time scale. The second part of equation, (1-*P*(*Δt*))*AS*(*t*), applies the return value from the agent-based system *AS*(*t*) at the lower time scale.

Importantly, the global parameter *Δt*, together with the description of the process behavior *P*(*Δt*), can be interactively changed. This interactivity enables us to model and visualize polymerization processes across different time scales during the simulation.

### Agent-based system

An agent-based system is utilized to capture the stochastic motion characteristics of monomers and the binding processes. The agent-based system is defined as *AS*(*t*)={*a*,*b*,*c*,…} where *t* is a global time parameter and *a*,*b*,*c*,… are sets of different types, in our case molecules.

Each agent has the following attributes: position, orientation, velocity, angular velocity and type. Additionally we define a set of functions representing its *conditions*, *behaviors* and *triggers*. Behaviors define the agent’s actions, conditions constrain agents within spatial boundaries and triggers are functions that are conditionally executed. The behavior of agents is not limited only to physical behavior. In our agent-based system the behavior of the agents can be defined to generally illustrate the process or to realistically simulate the required behavior.

In our case we wanted to illustrate diffusion movement and the binding process. However, there is a large time scale difference between them. The diffusion movement of the molecules is much faster than the binding process. Moreover, the time distance, in the time scale of binding, between two binding processes is comparably large. Therefore the agent-based system applies two types of approximations to the monomer movement based on whether the goal is to visualize monomer movement or the overall binding process.

If the agent-based system is used to interactively visualize the binding process of a monomer, random walking is applied to approximate the diffusion [27]:


The new position of the agent *a*_*pos*_ is updated by the diffusion coefficient *D*, time delta *Δt* and normal random vector *ξ*. It would take a long time if we would stay in this time scale and wait for a new molecule to come to the binding site and bind. Therefore if there is no binding process to illustrate, the simulation fast-forwards to the next binding event. During this stage the molecules are moving so quickly, that there is no visual correlation of monomers between two time steps. In this stage the monomers’ position and orientation are calculated based on a random distribution.

It is important to point out, that our aim is to sufficiently correctly illustrate the effect of diffusion and binding, not to realistically reproduce it. The speed of the process of monomer binding can be interactively altered by the global parameter *Δt* that specifies the amount of time between two simulation steps.

### System of densities

Here, we consider the overall densities of all involved agents of the agent-based system. The SOD is defined as a set of functions *SOD*={*d*_*a*_,*d*_*b*_,*d*_*c*_,…}. Each function represents the density of an agent type over time.

**Figure 5:**
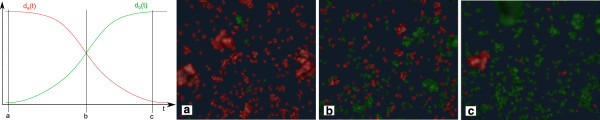
**An example of density-based steering possibilities for the agent-based system.** Two agent density functions change the number of agents in the agent-based system over time (from left to right). Panels show the state of the environment at the beginning **(a)**, in the middle **(b)**, and at the end **(c)** of the simulation.

Parallel to the other models, in every time step the SOD attempts to keep the number of agents ∥*a*∥ as close as possible to *d*_*a*_(*t*)×*V*, where *V* is the volume of the space in which the agents simulation runs. The user can steer the polymerization interactively by modifying the densities in the SOD. Figure 5 illustrates the behavior of the steering option.

## Implementation

Our implementation (Additional file 1) is based on the Unity3D framework [28]. This game engine is becoming increasingly popular, also within the bio-community [29]. Its simple *C#* programming interface provides fast prototyping possibilities and its efficient plugin system allows quick sharing of results, e.g., utilizing the Unity3D web-plugin.

### Visualization

Our polymerization visualization exploits 2D and 3D features of Unity3D. The number of molecules in both the agent-based system, as agents, and the L-system, as structural symbols, is in the order of thousands.

The geometrical representation of the molecules was generated with the VMD [30] software from PDB files. VMD is developed with NIH support by the Theoretical and Computational Biophysics group at the Beckman Institute, University of Illinois at Urbana-Champaign. The position of binding sites were also gathered from the PDB files and binding orientations were set manually from collected knowledge about the final appearance of the structures.

Each molecular mesh is obtained by means of the solvent excluded surface representation [31], which subsequently was simplified for performance reasons. This is because the generated raw molecular meshes are large (hundreds of thousands of triangles) and cause a performance bottleneck when using them. Thus, we sacrifice some geometric accuracy in order to devote more computational resources to the execution of our model.

**Figure 6:**
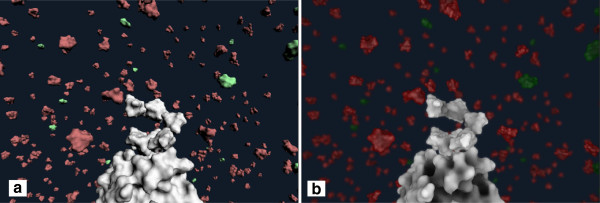
**A comparison between basic Unity3D diffuse rendering (a) and the additional use of screen space effects (b).**

**Figure 7:**
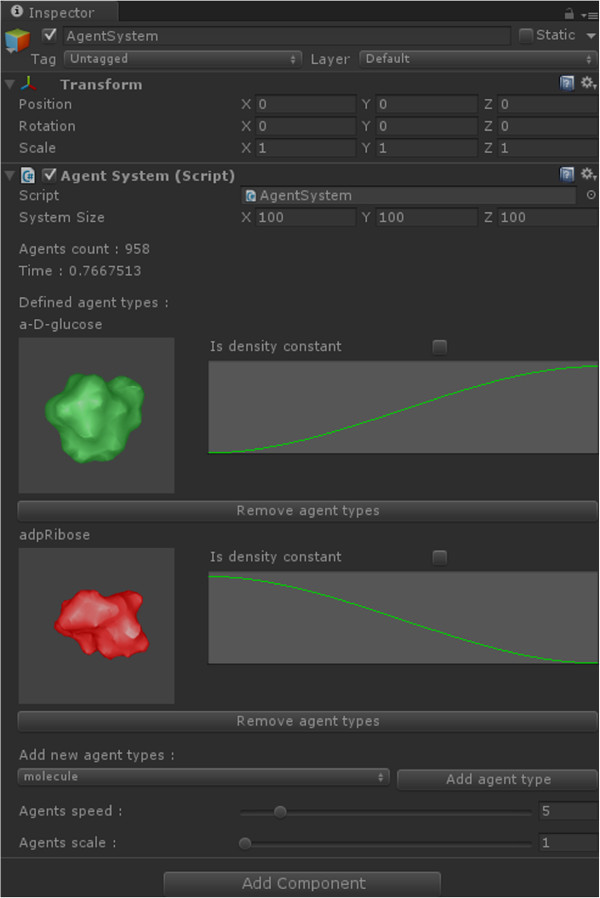
**An application screenshot depicting the Unity3D GUI for editing agents and their densities.**

We furthermore utilize screen space effects that add illustrative aspects to the eventual rendering (Figure 6). Namely, we perform an outline contour enhancement and screen space ambient occlusion [32].It is important to mention that all parameters regarding the shape and the visual molecular appearance can be adjusted by the user in the process of setting up the simulation through the Unity3D GUI (Figure 7).

### Interactivity

For the interactive exploration and experimentation with the simulation, our approach provides means to steer the camera, simulation, and L-system rules. These interactions have different impact on the simulation and can be categorized as follows:

- *Viewing interaction.* This category encompasses the interactions which affect the viewing parameters of the camera. The most important operation in this category is to look at the structures of the simulation from side to side, to zoom in and out to see details and to move with camera around and explore the environment.
- *Simulation steering.* Interactions from this category changes the parameters of simulation and have direct impact on it. However they are not meant to change the global behavior of the modeled process.
- An important interactive tool of the simulation is steering the count of molecule types during the simulation with the help of the SOD. Furthermore, it is possible to add new type or remove an existing type of molecule.
- Additionally, multiple temporal scales can be explored by changing the value of the global parameter *Δt*, which controls the speed of monomer movement during the binding process and also controls the switching between the time scales.
- *L-system control.* L-system rules can be added, changed or removed while the simulation is suspended. For example, the user can pause the simulation, and increase the probability of branching of the structure, by increasing the probability of the branching rule and decreasing the probability of the growing rule.

## Examples

Examples of naturally occurring polymers are DNA, proteins, glycogen, starch and poly-ADP-ribose. The structure of polymers is important for their physical properties, for example solubility [33]. This can be exemplified by looking at the properties of glucose polymers. Starch is a carbohydrate used to store energy in plants. It consists of two types of molecules, amylose and amylopectin. Amylose is composed of linear chains of glucose monomers and is insoluble in water, while amylopectin is composed of branched chains of glucose monomers, and is soluble in water. Polymers that contain one type of monomer are referred to as homopolymers, while polymers containing more than one type of monomer are referred to as heteropolymers. The DNA and proteins are made up of four and 20 monomers, respectively, hence are examples of heteropolymers. Glycogen, starch and poly-ADP-ribose are examples of homopolymers.

**Figure 8:**
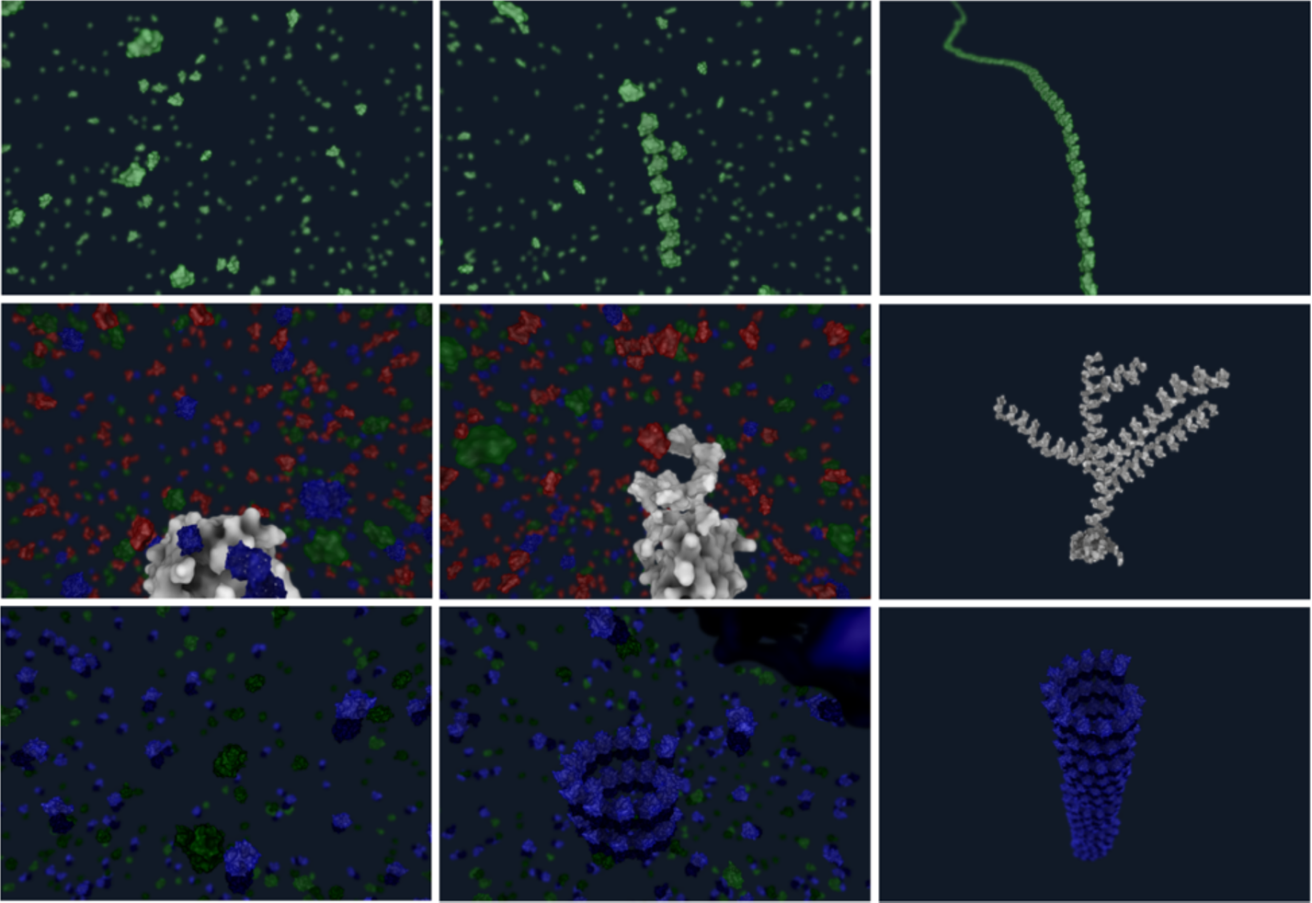
**An example of three polymerization processes: cellulose (1. row), poly-ADP ribose (2. rows) and microtubule (3. row).** Column-wise, the ordering (from left to right) represents the start of simulation, illustration of processes, and final structure.

Here we model reactions of glucose to form cellulose, ADP-ribose to form poly-ADP-ribose, and the creation of microtubules as examples of different types of bio-polymer architecture and composition. The results of our method are shown in Figure 8 (or Additional file 2). Our modeling approach and interactive simulation provides a visual environment for helping users (e.g., students) to understand these processes.

### Cellulose

Cellulose is an important structural component of plant cell walls and is one of the most common organic polymers on the planet [34]. It is made up of long unbranched chains of D-glucose, that are joint together by beta-1,4 glycosidic bonds. The length of the polymers may vary from a few hundred to thousands of monomers. Each D-glucose monomer is rotated by 180 degrees compared to the previous monomer in the chain. Parallel chains of cellulose may bind to each other to form secondary structures with various degrees of order. All of this results in fibers with various properties, and much research in the last 100 years has gone into understanding how this can be exploited.

Cellulose represents an example for the creation of linear homopolymers. In this example, we have molecules of D-glucose floating around in the environment. The polymer, and its creation, is expressed in the L-system with the symbolic alphabet *α*={*m*,*g*,*C*(*growth*),*ϵ*}. Where *m* is the structural symbol representing D-glucose, *g* is the binding symbol specifying that the next structure in the line will be placed above carbon 4 of D-glucose and rotated by 180 degrees. Lastly, *C*(*growth*,*Dglucose*,*t*,*r*) is a communication symbol specifying the process of growth by binding a new agent of type D-glucose to the structure with the process time *t* and the current process result *r*.

The rules from Appendix 1 were used for this example. The first rule *p*_1_ dictates, that if the result *r* of the symbol *C* is non-empty then the structure is extended by a new subunit *m* with position and rotation defined by *g* and on the end of this structure starts a new process of growing *C*(*growth*,*Dglucose*,0,∅).The mesh representation of the D-glucose molecule was exported from PDB with the VMD software. An outcome of the modeled cellulose polymerization is shown in the first row of Figure 8, where D-glucose molecules are visualized with green material.

### poly-ADP ribose

ADP-ribose is formed by cleaving Nicotinamide adenine dinucleotide (NAD) to form Nicotinamide and ADP-ribose. The ADP-ribose units may be attached to a variety of proteins, which create various signaling events in a cell [35]. Some of the events are triggered by attaching single ADP-ribose units, while other events are triggered by building ADP-ribose polymers on proteins. One event dependent on ADP-ribose polymers is NAD-dependent DNA repair. Single-strand breakage (SSB) or double-strand breakage (DSB) can potentially be very harmful to a cell unless properly repaired. Poly (ADP-ribose) polymerase (PARP) is an enzyme found in close proximity to the DNA, and is activated by SSB and DSB. It binds to the damaged site to protect the DNA ends, until the repair enzymes are in place. Once attached to the DNA, PARP auto-modifies itself by cleaving NAD molecules and attaching the resulting ADP-ribose monomers to a growing ADP-ribose polymer on itself. The final poly-ADP-ribose structure contains about 200 monomers with about 20-25 monomers per branch. ADP-ribose is negatively charged. This helps to recruit proteins involved in the DNA repair to the site. Since DNA is also negatively charged the growing tree will in addition pull PARP off the DNA, due to electrostatic forces. This makes room for the DNA repair enzymes to come in and repair the damaged site [35].

Poly-ADP-ribose represents an example for the creation of branched homopolymers. In the agent-based simulation, we have agents for NAD and other molecule types. The L-system alphabet *α*={*m*,*g*,*b*,*C*(*grow*),*C*(*branch*),*ϵ*} is composed of the structural symbol of ADP-ribose *m*, binding symbols *g* and *b*, where *b* is the beginning of a branch in the structure and *g* is the continuation of the branch. The communication symbols *C*(*grow*) and *C*(*branch*) describe the growing and branching processes.

For the polymerization of poly-ADP ribose the production rules from Appendix 2 were used. The development starts with the initial growing process *C*(*grow*,*NAD*,*t*,*r*). Rules *p*_1_ and *p*_2_ control the growth of the structure and the probability of starting the process of branching. When the branching process is finished, *p*_3_ creates the new branch and initiates its growth. Rules *p*_4_ and *p*_5_ are aging rules, meaning that if the process is not finished by the time *t*_*max*_, it will be terminated. The creation of poly-ADP ribose is shown in the second row of Figure 8. The NAD is visualized with red material. As soon as the NAD is processed and as ADP-ribose is attached to the structure, the color of the molecule is changed from red to white. The other molecules in the environment are colored with green and blue material.

### Microtubules

Microtubules are long tubular polymers that are involved in a number of important cellular processes. They are found in the cytoplasm of eukaryotic cells, where they act as part of the structural framework that determines cell shape and cell movements. Microtubules also have important roles in the cell division and act as a railway system for intracellular transport. The microtubule polymers consists of repeating units of a globular protein called tubulin. Tubulin is a dimer which is made up of two polypeptides, called alpha and beta tubulin. A microtubule generally consists of 13 protofilaments [36] assembled around a hollow core. The protofilaments are composed of arrays of tubulin dimers, that are arranged in parallel. The assembly and disassembly of microtubules is highly dynamic. A detailed review of these processes can be found in the work of Akhmanova et al. [37].

From the structural, and content point of view, the microtubule represents an example of linear heteropolymers. For this example, the agent-based system contains agent types of tubulin and background molecules. The Tubulin agent is composed of coupled agents of alpha tubulin and beta tubulin. The L-system has an alphabet *α*={*a*,*b*,*v*,*h*,*C*(*grow*)*ϵ*}, where *a* and *b* are structural symbols of alpha tubulin and beta tubulin. The binding symbols *v* and *h* define the binding between the alpha and the beta tubulin, which creates the inner structure of the tubulin dimer, and the binding between two neighboring dimers. The process of growing the structure is described by the communication symbol *C*(*grow*).

The corresponding rules from Appendix 3 define the overall microtubule creation. The rule *p*_1_ attaches the monomers of the tubulin dimer (alpha and beta tubulin) to the structure and continues the growing at the end of the structure. The third row of Figure 8 shows different stages of the development, where the new dimer is always connected to the end of the spiral. The polymerization of microtubules, as described in [37], is believed to occur in sheets which fold into the circular structure. Our visualization differs from this description (tubular geometry is produced directly) since we do not model the forces necessary to complete the folding process.The microtubule example is shown in the third row of Figure 8. The tubulin dimer consists of alpha tubulin molecule, in light blue, and beta tubulin, in dark blue.

### Synthetic, non-biological showcase

Our approach can model the emergence of more complex structures than what was described in the previous examples.

In this example, we demonstrate the creation of complex branching patterns in an overall structure with different types of subuints. The structure starts with one type of subunits, for example spheres, which creates helices and have also branches of the same type and secondary structuring. The main branch ends with the star branching. These branches can be completely different than main branch. In our demonstration these are composed of two periodically altering types, cubes and cylinders, are linear and doesn’t create helical secondary structure.

The L-system rules for the overall process are defined in Appendix 4. Rules *p*_1_,*p*_2_ and *p*_3_ are responsible for the growing of the main branch and initiating the growth of other branches. The rules *p*_4_,*p*_5_ set the creation and growth of the branches from the main branch. Lastly the rules *p*_6_,*p*_7_,*p*_8_,*p*_9_ manage the creation of the star architecture on the top of the structure, stopping the growth of the main branch. These also manage the growth of the star branches in a way that two types of subunits are placed periodically.

## Evaluation

We have discussed the presented examples of our system with two experts in the field of biology and bioinformatics and one expert from the molecular illustration field. The demonstration of our system was presented as a video showing animations of the mentioned biological examples. Also the interactivity of the system was presented by video demonstrate the effect of parameter changes. For every example, we provided the biological explanation and afterwards the users observed the system for several minutes.

Professor Mathias Ziegler, expert in the field of biology, was impressed by the outcome of our approach. He mentioned that the system could generate several proto-structures and model energy requirements for the reactions. With this extension he could imagine that it may be used for the generation (and even for the testing) of hypotheses for molecular phenomena that require spatial information.

For example, one question to which our system, with the suggested extensions, could possibly bring an answer is, what is the ideal branching percentage for the best coupling of glycogen is. Since we can change the parameters of the L-system rules at runtime, users can interactively experiment with the probability of rules and study the emergent branching structure.

He particularly appreciated the system of density layer for the control of the molecule counts during the simulation and the interactive change of modeling rules. In his opinion, the outcome of our work can be used for teaching purposes. Especially, he was impressed by the capability of our system to create complex structures simply from information of the geometrical representation of subunits, their binding sites, and simple rules.

Another expert, Assoc. Prof. in Molecular Bioinformatics, suggested that we could show the outcome of our system in the context of examples of multimeric structures, especially when it comes to complex formation. Additionally, she pointed out that all polymer formations are catalyzed by enzymes and in many cases this is what determines the later structure as well as the speed of the assembly. With this addition we could provide better biological understanding of these processes in the context of teaching. She also pointed out that with further extensions of the work we could be able to bring answers to some unsolved questions in the field of polymer synthesis. Another aspect in the context of polymerization is the possibility that a local depletion of pre-cursors might be the factor that limits the chain length.

We also discussed our approach with a professional illustrator. She pointed out the importance of having a system for generating a complex, dynamic, and accurate biological scene in a time and cost-efficient manner. Being able to easily generate dynamic, accurate, and aesthetically pleasing molecular scenes is extremely beneficial for animators and scientific filmmakers.

From a biomedical animation point of view, she praised the system as a quick, easy to use, and flexible tool for generating good quality and aesthetically pleasing images. However, she was missing more control over rendering styles and lighting. While she saw the system as an excellent start, being able to bring these dynamic systems directly into 3D animation software would be, in her opinion, ideal. Overall, she considered the biological scenes generated from this system useful for producing biological animations.

Many of the ideas of the domain experts, are good suggestions and will be considered in future work.

## Results and discussion

Our modeling system is composed of three main parts, i.e., the L-system with communication symbols, an agent-based system, and a system of densities. Their behavior and their interactions are determined by defining the agents’ behavior, and their numbers and by specifying the L-system’s alphabet and production rules.

We demonstrated the use of this modeling system in the context of several examples from molecular biology that capture the creation of different types of polymers. We found out that the proposed modeling and visualization system makes it possible to easily create, modify, and visualize models at different spatial and temporal scales. The simulations of the polymerization were fast enough to allow for interactive experimentation with the models.

**Figure 9:**
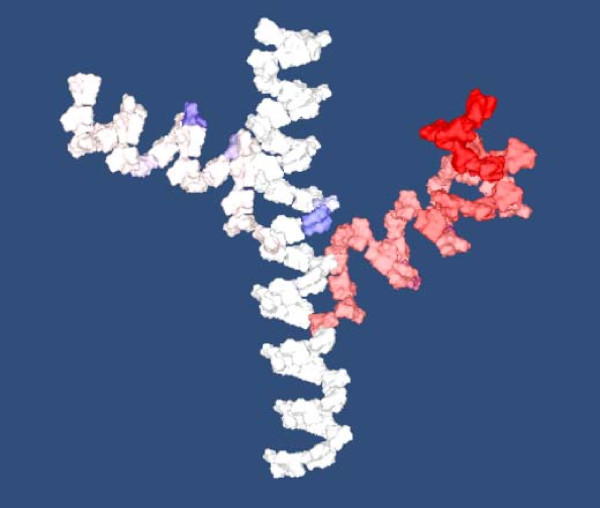
**An visualization example of branching uncertainty and branching probability factor in the resulting structure.** Branching uncertainty is accumulated using a Gaussian kernel centered at the probability threshold for branching and growing. It is visualized in the new branches as transition from white, no uncertainty, to red, high uncertainty. The blue color indicates the branching probability for cases when no new branch was created.

In the process of developing this model we became increasingly aware of the lack of information about the creation of polymer structures. This opens a door for the possibility to use our approach for hypothesis generation or at least as a testing environment for the study of polymerization.We also found out, that the visualization part of our approach can be extended to encode additional interesting information about the simulation. For instance the uncertainty of the branching probability of the structure has considerable impact on the resulting geometrical structure and, therefore, it is interesting to explore its influence. These information is stored in the L-system symbols. Figure 9 provides a visualization of the created structure with the values of branching uncertainty (white to red) and branching probability (white to blue). Our approach flexibly supports the study of this and similar properties of the model and can therefore has the potential to provide valuable insights beyond the generated geometric structures.

Limitations of our current implementation include the absence of modeling third parties in the process, for example enzymes. Additionally, the rules of the L-system are not context-sensitive, meaning that we are unable to model sub-processes, which depend on neighborhood information in the structure. Another challenge is the integration of rigid body simulation and force fields into the resulting structure, simulating biologically feasible, dynamic behavior and processes dependent on them, as was pointed out in example of microtubules polymerization.

## Conclusions

We have presented a novel modeling approach that is capable of illustrating polymer emergence within a filled environment of stochastically moving molecules. Our approach is a fusion of three systems combining the complementary advantages of three distinct modeling approaches. The resulting system can model, simulate, and interactively visualize emergence in a stochastic environment at different time scales. Also, it satisfies all the properties, which were identified for proper modeling of the emergence phenomena.

We demonstrated the possibilities of the model in examples of polymerization of linear and branched polymers with one or several types of monomers. However, the fusion of models could also be potentially used in other applications, for example to model the emergence of coral reefs, bacterial cultures, or in fields outside of biology, e.g., for the procedural modeling of cities, growth of infrastructure, or emergence of crystals.


## Electronic supplementary material

Additional file 1: **Prototype unity project.** The ZIP file comprises a prototype project with example scenes. Prototype project can be opened by Unity editor. Which can be downloaded from http://unity3d.com/unity/download web page. Detailed description of the examples and prototype usage is available from http://www.ii.uib.no/vis/projects/physioillustration/research/interactive-molecular-illustration.html. (ZIP 6 MB)

Additional file 2: **Video demonstration.** Video showing the interactivity of the system and the illustrative visualization of polymer emergence through examples of Cellulose, PARP, Microtubules and artificial more complex showcase. (MP4 17 MB)

## Abbreviations

ADP: Adenosine diphosphate
DNA: Deoxyribonucleic acid
DSB: Double-strand breakage of the DNA
NAD: Nicotinamid adenin dinucleotide
NIH: National Institutes of Health
PARP: poly (ADP-ribose) polymerase
PDB: Protein Data Bank
SOD: System of densities
SSB: Single-strand breakage of the DNA
VMD: Visual molecular dynamics.
