# Supplementary figures and images for: Interactively illustrating polymerization using three-level model fusion
Source: BMC Bioinformatics. 2014 Oct 14;15(1):345. doi: 10.1186/1471-2105-15-345 (PMC4287442; doi:10.1186/1471-2105-15-345)

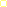

Supplement: Supplementary file 1 — Additional file 1: Prototype unity project. The ZIP file comprises a prototype project with example scenes. Prototype project can be opened by Unity editor. Which can be downloaded from http://unity3d.com/unity/download web page. Detailed description of the examples and prototype usage is available from http://www.ii.uib.no/vis/projects/physioillustration/research/interactive-molecular-illustration.html. (ZIP 6 MB) [file 12859_2014_6668_MOESM1_ESM.zip › UnityProject/Assets/Resources/Images/boxActive.png]

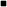

Supplement: Supplementary file 1 — Additional file 1: Prototype unity project. The ZIP file comprises a prototype project with example scenes. Prototype project can be opened by Unity editor. Which can be downloaded from http://unity3d.com/unity/download web page. Detailed description of the examples and prototype usage is available from http://www.ii.uib.no/vis/projects/physioillustration/research/interactive-molecular-illustration.html. (ZIP 6 MB) [file 12859_2014_6668_MOESM1_ESM.zip › UnityProject/Assets/Resources/Images/boxBackground.png]
